# Late malfunction of subcutaneous implantable cardioverter-defibrillator in a patient with advanced emphysema

**DOI:** 10.1016/j.hrcr.2024.04.006

**Published:** 2024-04-18

**Authors:** Hafez Golzarian, Robbie J. Meyer, Amy K. Carder, Fayaz A. Hakim

**Affiliations:** ∗ Internal Medicine Residency Program, Mercy Health St. Rita’s Medical Center, Lima, Ohio; † Heart Rhythm Services, Division of Cardiovascular Diseases, St. Rita’s Medical Center, Lima, Ohio

**Keywords:** Implantable cardioverter-defibrillator, Sudden cardiac death, Emphysema, Defibrillation, COPD, Praetorian scoring

**Key Teaching Points**

• Air entrapment from progressive pulmonary emphysema can lead to late subcutaneous implantable cardioverter-defibrillator (S-ICD) malfunction.
• Proper preimplant screening including a qualitative computed tomography scan of lungs should be performed in patients with severe emphysema with an indication for ICD to assess the severity of air entrapment, prior to considering them for S-ICD implantation.
• Patients with emphysema and S-ICD require close monitoring for S-ICD malfunction and air entrapment from progression of emphysema.

## Introduction

Implantable cardioverter-defibrillators (ICD) are highly effective in treating life-threating ventricular arrhythmias and are commonly implanted in patients at risk of sudden cardiac death. Successful defibrillation by an ICD depends on its ability to deliver shocks that exceed defibrillation thresholds. In a properly implanted and normal-functioning ICD system, extracardiac conditions (eg, pneumothorax, pleural effusion, excessive soft tissue) can increase shock impedances and divert the shock pathway, resulting in defibrillation failure. Among patients with transvenous ICD (TV-ICD), persistent elevation in high-voltage impedance, defibrillation threshold testing (DFT) failure, and ICD shock failures have all been reported in patients with pneumothorax, air pockets around the pulse generator, and subcutaneous emphysema.^1^, ^2^, ^3^ Undersensing of ventricular fibrillation and oversensing leading to inappropriate shocks secondary to air entrapment has been reported with subcutaneous ICDs.^4^^,^^5^ We present a unique case of late S-ICD malfunction owing to air entrapment from disease progression in a patient with advanced emphysema. This case elucidates the importance of proper screening and work-up of patients with a history of emphysema prior to considering them for S-ICD implantation.

## Case report

**Figure 1:**
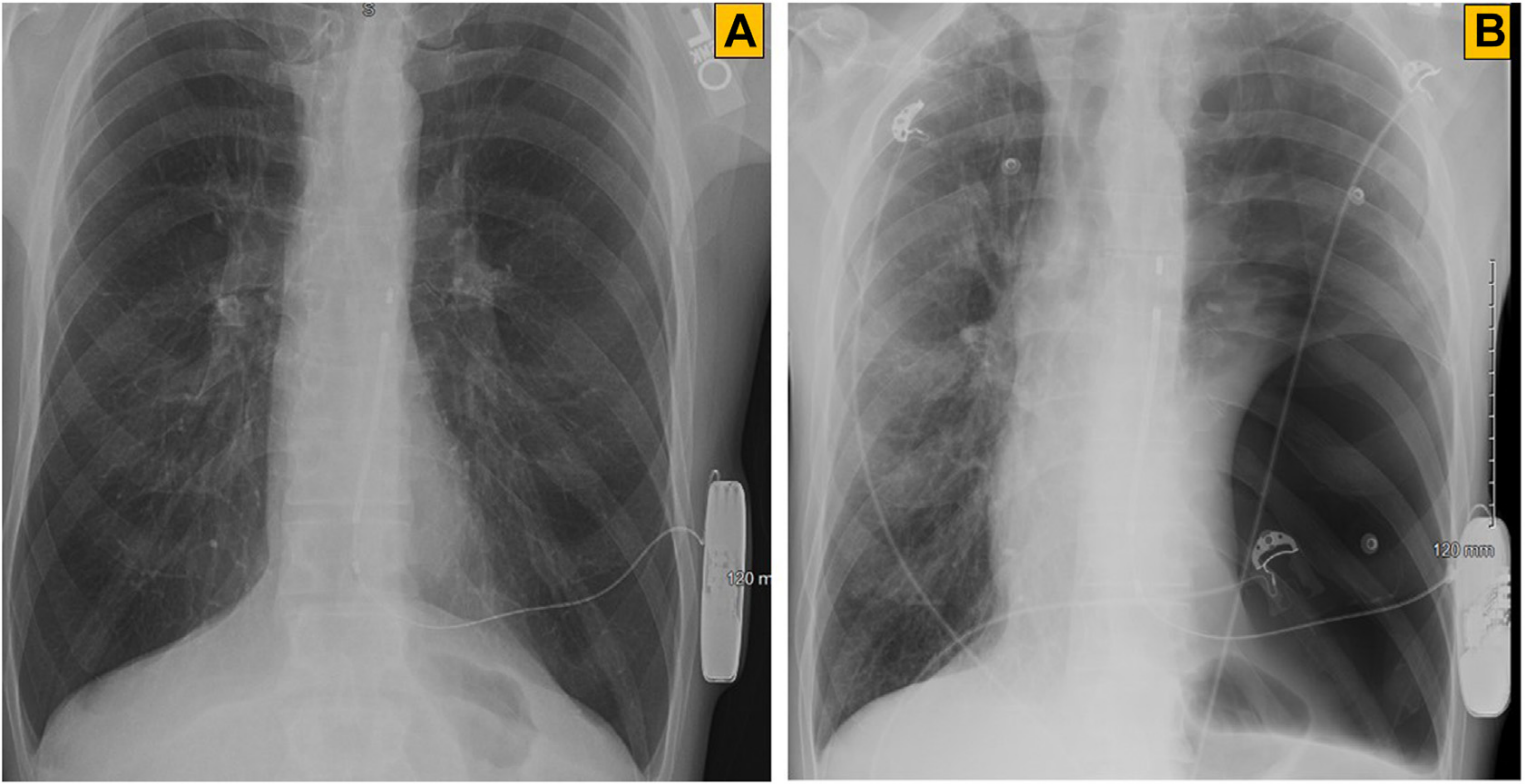
Chest radiographs, posteroanterior views. **A:** Obtained at subcutaneous implantable cardioverter-defibrillator implantation, showing hyperinflated lungs with radiolucent areas bilaterally, more pronounced on left side. **B:** At device malfunction, showing progressive disease, traction bronchiectasis, and expansion of emphysematous bullae.

**Figure 2:**
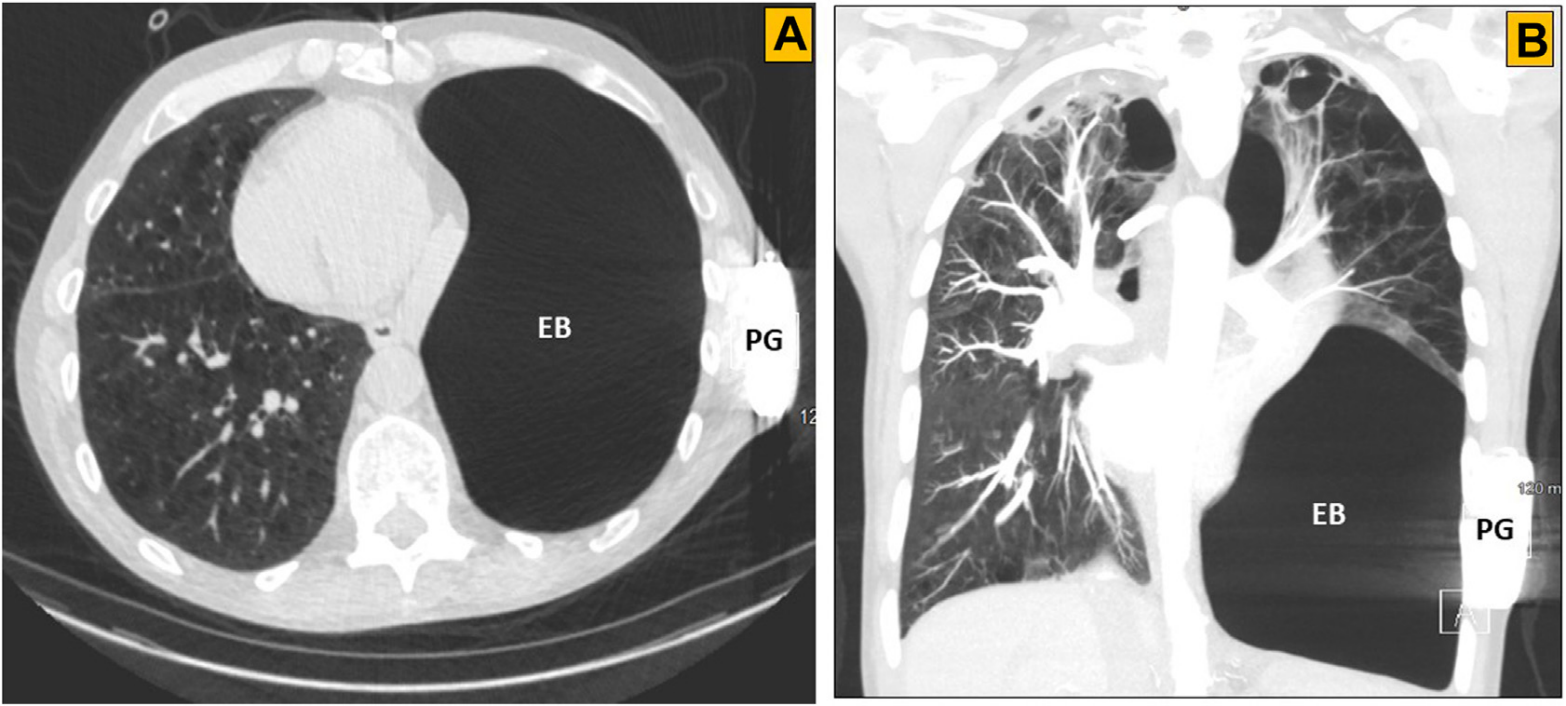
Computed tomography scan of the chest showing advanced emphysema, traction bronchiectasis, and a large emphysematous bulla occupying entire left lower hemithorax in shock path. **A:** Coronal view. **B:** Sagittal view. EB = emphysematous bulla; PG = pulse generator.

**Figure 3:**
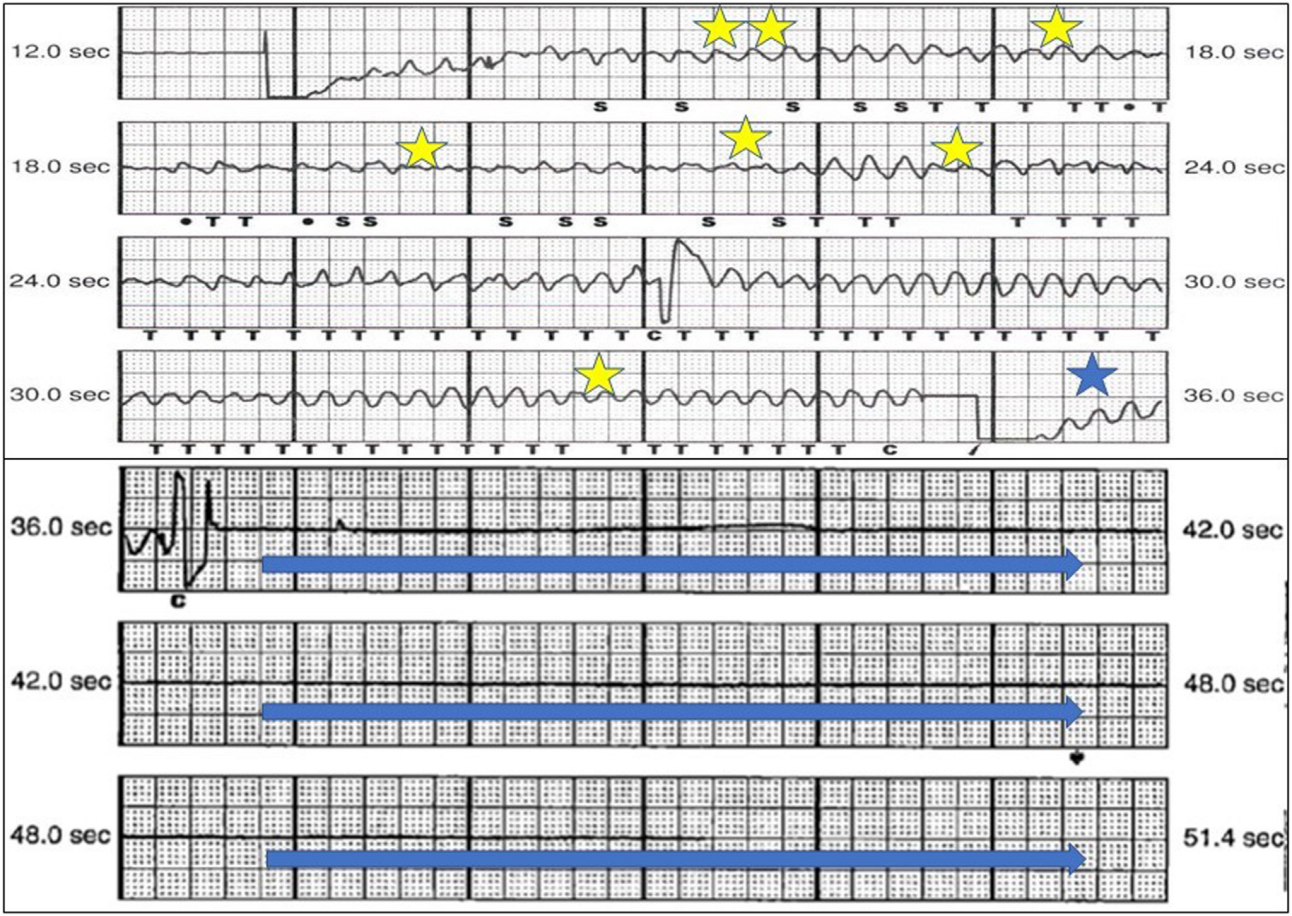
Subcutaneous implantable cardioverter-defibrillator tracing at defibrillation threshold testing showing sensing dropouts (*yellow stars*), failed shock (*blue star*), and complete loss of sensing (*blue arrows*).

**Supplemental Figure 1:**
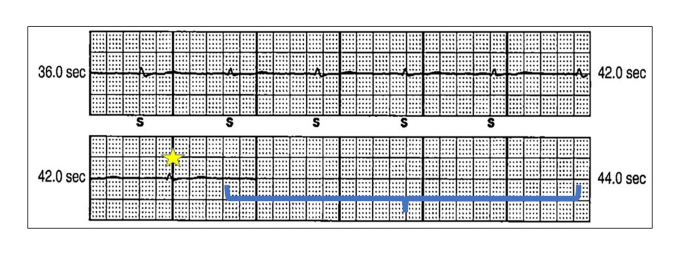
S-ICD tracing showing small R wave amplitude, Undersensing (yellow star) and failure to register R wave (blue parenthesis).

**Supplemental Figure 2:**
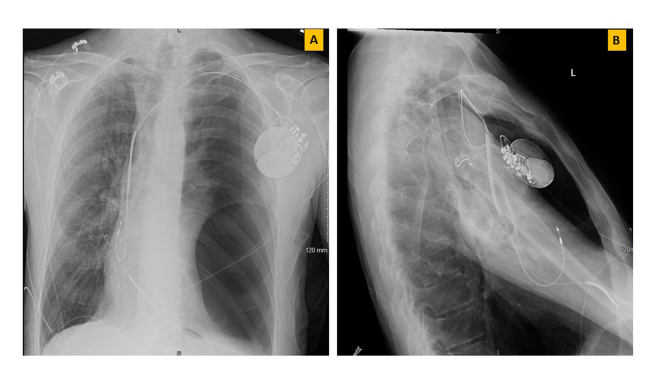
Chest radiography PA (A) and lateral (B) views showing transverse ICD systems with emphysematous bulla away from the shock vectors.

A 57-year-old male with history of witnessed sudden cardiac arrest owing to ventricular fibrillation underwent a successful subcutaneous ICD (S-ICD) (Boston Scientific, Marlborough, MA) implantation for secondary prevention of sudden cardiac death at an outside medical institution 6 years ago. During DFT at implantation, the first 80 J shock was successful and high-voltage shock impedance was 86 ohms in primary sensing vector. Postimplant chest radiographs showed appropriate locations of the pulse generator and defibrillation electrode. Radiolucency within the lung parenchyma adjacent to the S-ICD pulse generator and defibrillation coil was notable as well (Figure 1A). Evaluations at the time of the patient’s cardiac arrest, including routine laboratory tests, electrocardiogram, and echocardiogram, were unremarkable. Coronary angiography showed patent coronary arteries. The patient’s past medical history was significant for 35 pack-years of smoking and smoking-related advanced emphysema, for which he had undergone lung volume reduction surgery 3 years prior. He had tested negative for α1-antitrypsin deficiency PiZZ phenotype. He was on continuous home oxygen and inhaled long-acting beta-2 agonist and steroid combination. Family history was negative for malignant arrhythmia, cardiomyopathy, or sudden cardiac death. The patient received 2 appropriate shocks from his device for fast ventricular tachycardia during the first year of device implantation and was arrhythmia free on sotalol since then. A year ago, during a routine device check, he was noted to have intermittent loss of sensing and failure to register R waves (Supplemental Figure 1). The chest radiograph (Figure 1B) and a computed tomography (CT) scan (Figure 2) showed advanced emphysema, traction bronchiectasis, and a large emphysematous bulla compressing the lung and shifting the mediastinum to the right. The S-ICD pulse generator and defibrillator electrode were in good locations. A DFT and pulse generator change was recommended, as his device was near elective replacement indicator. In the electrophysiology laboratory, general anesthesia was administered. The patient was prepped and draped in a standard sterile manner using 4-piece surgical drape. Low-voltage shock impedance with 10 J energy in sinus rhythm was 120 ohms. Ventricular fibrillation was induced by 40 Hz pacing. After initial sensing dropouts, the sensing and charging were appropriate, but 65 J shock from the device was unsuccessful (Figure 3). High-voltage shock impedance was 130 ohms. Following the delivery of shock, complete loss of sensing was noted (Figure 3). A total of three attempts to defibrillate with 360 J external shocks through anterior and lateral chest wall defibrillator pads were unsuccessful. The patient’s chest was exposed and another external shock at 360 J with paddles applied anteriorly and posteriorly was successful in restoring sinus rhythm. The S-ICD system was explanted, followed by implantation of a conventional transvenous dual-chamber dual-coil ICD (Supplemental Figure 2). DFT was successful with the first 36 J shock and high-voltage shock impedance was 60 ohms. At 24 months follow-up, the patient was arrhythmia-free on sotalol. The sensed R wave was 11 mV, capture threshold 1 V @ 0.4 ms, pace impedance 660 ohms, and high-voltage shock impedance 64 ohms.

## Discussion

We report a case of late S-ICD malfunction owing to disease progression and air entrapment in a patient with advanced emphysema in whom DFT at implantation was successful and high-voltage shock impedance acceptable despite evidence of significant lung disease.

The S-ICD system is noninferior to conventional TV-ICD, with a probability of successful termination of malignant ventricular arrhythmia approaching >95%.^6^, ^7^, ^8^ The utilization of the S-ICD has eliminated acute and chronic endovascular complications associated with conventional TV-ICD systems (eg, chronic venous occlusions, endovascular infections, and potentially fatal complications during indicated lead extractions).^9^ S-ICD is therefore an attractive alternative to TV-ICD for prevention of sudden cardiac death from malignant ventricular arrhythmia in the absence of indications for pacing for bradyarrhythmia, cardiac resynchronization, or treatment of antitachycardia pacing-responsive ventricular tachycardia.^10^ Following U.S. Food & Drug Administration approval in 2012, the use of S-ICD was initially limited to younger patients with mild or no structural heart diseases and fewer comorbidities. The efficacy and safety of S-ICD systems in older patients with structural heart disease and more comorbidities has been reported to be noninferior to TV-ICD in a large representative cohort.^11^^,^^12^ Hence, it is now being implanted frequently in older patients with structural heart diseases and more comorbidities, especially in those with increased risk of infection (eg, immune compromised) or in whom preservation of venous patency is desired (eg, chronic kidney disease).

In comparison to the TV-ICD, the S-ICD system is exclusively extravascular and extracardiac, thus making its functionality potentially more vulnerable. Any amount of extra insulation in the form of air, fluid, or tissue in the shock pathway may have a significant impact on the proper functioning of the device. Hence, appropriate positioning and approximation of the pulse generator and defibrillation electrode to the left lateral rib cage and to the sternum, respectively, are crucial for successful defibrillation in the S-ICD system. For the same reasons, in appropriately selected patients who pass preimplant screening, a DFT at the time of S-ICD implantation is routinely performed as a class I recommendation to ensure proper sensing of ventricular fibrillation and confirm successful defibrillation.^9^^,^^13^ A shock impedance of <90 ohms has been shown to correlate with a defibrillation threshold success rate of >95%.^14^ Alternatively, R-wave synchronous shock with high (65 J) or low (10 J) energy in sinus rhythm has been shown to assess the overall system integrity in patients in whom DFT is not performed for safety reasons.^13^ If any shock administration is deemed to be inappropriate owing to safety concerns, a noninvasive postimplant Praetorian score can be used as an alternative to DFT and shock impedance testing, using 3 independent determinants: (1) position of the pulse generator with respect to left mid axillary line, (2) thickness of the subcutaneous fat between the pulse generator and the thoracic wall, and (3) the number of coil widths of fat tissue between the S-ICD coil and the sternum. A Praetorian score of <90 predicts a low risk and a score ≥150 predicts a high risk of shock failure.^14^ Hence, many S-ICD implanters are omitting DFT and are relying on sinus rhythm low-voltage shock impedances or sometimes on postimplant Praetorian scoring.

Emphysema is a distal airspace disease, characterized by destruction of lung parenchyma with loss of elastic tissue without fibrosis, resulting in airway obstruction and air trapping. Emphysema is a progressive disease, even after cessation of smoking. The presence of emphysema, defined by CT scan imaging of lungs, among smokers is associated with progression of emphysema in all GOLD stages, regardless of presence or absence of symptoms and spirometry abnormalities.^15^

Our patient had evidence of air trapping in the shock pathway, which was overlooked as a potential cause of shock failure. A preimplant quantitative CT scan of the chest might have allowed better assessment of disease severity and air entrapment and prompted against S-ICD implantation. A successful DFT with a high-voltage shock impedance of <90 ohms and successful treatment of 2 episodes of fast ventricular tachycardia postimplant were reassuring but did not reliably predict long-term efficacy of S-ICD owing to his progression of emphysema. Progressive air trapping and expansion of emphysematous bullae resulted in sensing abnormalities, increased high-voltage shock impedance, and DFT failure, necessitating exchanging of the S-ICD system with a conventional TV-ICD.

Emphysema is not an uncommon comorbidity among older patients undergoing ICD implantations. Such patients should undergo careful evaluation before they are deemed candidates for S-ICD, including a quantitative CT scan of the chest to assess for the presence and severity of air trapping, especially in the lower lobe of the left lung, which could be subtle and easily overlooked on plain radiograph of the chest. A normal DFT and high- or low-voltage shock impedance <90 ohms at the time of implant in patients with mild or even moderate emphysema may not necessarily predict long-term success from progression of emphysema, especially in patients with bullous disease. A pneumothorax resulting from rupture of small bulla, which may be even remote to the shocking vector, can have similar detrimental effects in S-ICD functions. Patients with severe or advanced emphysema and those with bullous disease, especially involving the lower lobe of the left lung, should be considered for conventional TV-ICD systems. Issues related to progressive emphysema may occur in TV-ICD, but options of adding a left subclavian or coronary sinus defibrillation coil in such systems may allow feasible troubleshooting. Extravascular ICDs such as the Aurora EV-ICD System (Medtronic, Dublin, Ireland), a new addition to extravascular and extracardiac ICD systems, potentially can have similar challenges and are also better avoided in such patients. Patients with severe and advanced emphysema and S-ICD should be carefully monitored for device malfunction and emphysema progression. A CT scan of the chest should be performed after lung volume reduction surgery for assessing worsening air trapping, especially in the path of the shocking vector.

## Conclusion

Emphysema is a progressive disease that may result in significant air trapping from bullae expansion and pneumothorax and causes S-ICD failure. Proper preimplant screening including a quantitative CT scan of the chest should be incorporated in the assessment process for the candidacy for S-ICD systems in patients with emphysema. Should such patients undergo S-ICD implantations, close monitoring for S-ICD malfunction and disease progression will allow timely and appropriate interventions.

## Disclosures

The authors have no financial or personal relationships to disclose.

## References

[1] Cohen T.J., Lowenkron D.D. (1998). Defibrillation threshold and pneumothorax. Pacing Clin Electrophysiol.

[2] Luria D., Stanton M.S., Eldar M., Glikson M. (1998). Pneumothorax: an unusual cause of ICD defibrillation failure. Pacing Clin Electrophysiol.

[3] Gibino F., Vitali F., Malagu M. (2023). Subcutaneous emphysema after spontaneous pneumothorax: a rare cause of persistent increase of shock impedance in an implantable cardioverter-defibrillator. HeartRhythm Case Rep.

[4] Adduci C., Spadoni L., Palano F., Francia P. (2019). Ventricular fibrillation undersensing due to air entrapment in a patient implanted with a subcutaneous cardioverter defibrillator. J Cardiovasc Electrophysiol.

[5] Lee S., Souvaliotis N., Mehta D., Suri R. (2017). Inappropriate shock in a subcutaneous cardiac defibrillator due to residual air. Clin Case Rep.

[6] Knops R.E., Olde Nordkamp L.R.A., Delnoy P.H.M. (2020). Subcutaneous or transvenous defibrillator therapy. N Engl J Med.

[7] Gold M.R., Lambiase P.D., El-Chami M.F. (2021). Primary results from the Understanding Outcomes With the S-ICD in Primary Prevention Patients With Low Ejection Fraction (UNTOUCHED) trial. Circulation.

[8] Boersma L.V., El-Chami M.F., Bongiorni M.G. (2019). Understanding Outcomes With the EMBLEM S-ICD in Primary Prevention Patients with Low EF study (UNTOUCHED): clinical characteristics and perioperative results. Heart Rhythm.

[9] Al-Khatib S.M., Stevenson W.G., Ackerman M.J. (2018). 2017 AHA/ACC/HRS guideline for management of patients with ventricular arrhythmias and the prevention of sudden cardiac death: a report of the American College of Cardiology/American Heart Association Task Force on Clinical Practice Guidelines and the Heart Rhythm Society. J Am Coll Cardiol.

[10] Friedman D.J., Qin L., Parzynski C. (2022). Longitudinal outcomes of subcutaneous or transvenous implantable cardioverter-defibrillators in older patients. J Am Coll Cardiol.

[11] Gold M.R., El-Chami M.F., Burke M.C. (2023). Postapproval study of a subcutaneous implantable cardioverter-defibrillator system. J Am Coll Cardiol.

[12] Ben Kilani M., Jacon P., Badenco N. (2023). Defibrillation testing during S-ICD implantation: How relevant? Results from a multicenter study. EP Europace.

[13] Amin A.K., Gold M.R., Burke M.C. (2019). Factors associated with high-voltage impedance and subcutaneous implantable defibrillator ventricular fibrillation conversion success. Circ Arrhythm Electrophysiol.

[14] Quast A.F.B.E., Baalman S.W.E., Brouwer T.F. (2019). A novel tool to evaluate the implant position and predict defibrillation success of the subcutaneous implantable cardioverter-defibrillator: the PRAETORIAN score. Heart Rhythm.

[15] Pompe E., Moore C.M., Firdaus A.A. (2021). Progression of emphysema and small airways disease in cigarette smokers. Chronic Obstr Pulm Dis.

